# Relative importance of chemical attractiveness to parasites for susceptibility to trematode infection

**DOI:** 10.1002/ece3.4386

**Published:** 2018-08-13

**Authors:** Laura Langeloh, Otto Seppälä

**Affiliations:** ^1^ Institute of Integrative Biology (IBZ) ETH Zürich Zürich Switzerland; ^2^ Eawag Swiss Federal Institute of Aquatic Science and Technology Dübendorf Switzerland

**Keywords:** cercaria, chemical detection, host finding, host–parasite interactions, immune defense, parasite resistance

## Abstract

While the host immune system is often considered the most important physiological mechanism against parasites, precontact mechanisms determining exposure to parasites may also affect infection dynamics. For instance, chemical cues released by hosts can attract parasite transmission stages. We used the freshwater snail *Lymnaea stagnalis* and its trematode parasite *Echinoparyphium aconiatum* to examine the role of host chemical attractiveness, physiological condition, and immune function in determining its susceptibility to infection. We assessed host attractiveness through parasite chemo‐orientation behavior; physiological condition through host body size, food consumption, and respiration rate; and immune function through two immune parameters (phenoloxidase‐like and antibacterial activity of hemolymph) at an individual level. We found that, although snails showed high variation in chemical attractiveness to *E. aconiatum* cercariae, this did not determine their overall susceptibility to infection. This was because large body size increased attractiveness, but also increased metabolic activity that reduced overall susceptibility. High metabolic rate indicates fast physiological processes, including immune activity. The examined immune traits, however, showed no association with susceptibility to infection. Our results indicate that postcontact mechanisms were more likely to determine snail susceptibility to infection than variation in attractiveness to parasites. These may include localized immune responses in the target tissue of the parasite. The lack of a relationship between food consumption and attractiveness to parasites contradicts earlier findings that show food deprivation reducing snail attractiveness. This suggests that, although variation in resource level over space and time can alter infection dynamics, variation in chemical attractiveness may not contribute to parasite‐induced fitness variation within populations when individuals experience similar environmental conditions.

## 1 INTRODUCTION

By making up around 40% of the known species (Dobson, Lafferty, Kuris, Hechinger, & Jetz, 2008), parasites are a common selective force in nature (Hamilton, Axelrod, & Tanese, 1990). Most free‐living organisms are infected with parasites that induce fitness costs by reducing host survival (e.g. Brown, Brown, & Rannala, 1995; Martínez‐de la Puente et al., 2010) and fecundity (e.g. Hurd, 2001; Stirnadel & Ebert, 1997). As a response to such selection, hosts have developed defense mechanisms to prevent infections. Of these, the immune system is considered to be the most important physiological barrier against parasites (reviewed in Janeway, Travers, Walport, & Shlomchik, 2005). However, also other host characteristics may determine its susceptibility to infections (reviewed in Parker, Barribeau, Laughton, de Roode, & Gerardo, 2011; Seppälä, 2015). For instance, precontact mechanisms such as avoidance of infected individuals (Behringer, Butler, & Shields, 2006; Kiesecker, Skelly, Beard, & Preisser, 1999) as well as habitat and dietary choices (Hutchings, Gordon, Kyriazakis, & Jackson, 2001; Lefèvre et al., 2012) can greatly affect the risk of exposure to parasites. Therefore, such mechanisms could act as alternative targets for parasite‐mediated selection. However, the relative roles of pre‐ and postcontact mechanisms in determining host susceptibility are typically not understood.

Many parasites have free‐living larval stages that seek hosts in the environment (Combes, 1991). This involves movement toward suitable microhabitats based on cues such as gravity, temperature, and light intensity (e.g. Loy, Motzel, & Haas, 2001; Takahashi, Mori, & Shigeta, 1961). After reaching the proximity of potential hosts, parasites can use chemical cues released by the hosts to detect them (De Bruyn, De Ridder, Rigaud, & David, 2011; Haas, Haberl, Kalbe, & Körner, 1995; Hallem et al., 2011; Hertel, Holweg, Haberl, Kalbe, & Haas, 2006). The quantity and quality of chemical cues emitted by hosts can depend on their physiological characteristics and condition (Becker & Schmale, 1978; Körner & Haas, 1998), potentially leading to variation in host exposure to parasites. Although only little is known about the relative importance of such variation in determining host susceptibility, its impact on parasite infection dynamics can be high. For example, in the freshwater snail *Lymnaea stagnalis*, experimental manipulation of host resource level using feeding treatments affects its chemical attractiveness to trematode parasites as well as parasite infection success (Seppälä, Karvonen, Haataja, Kuosa, & Jokela, 2011; Seppälä & Leicht, 2015). More specifically, food‐limited snails harbor fewer parasites, which is due to reduced chemo‐orientation of parasite larvae toward them. These earlier studies indicate that variation in environmental conditions both over time and space can alter exposure to parasites and thus disease dynamics. They also suggest that host physiological condition is the underlying mechanism determining altered susceptibility. However, those studies do not test the importance of variation in host chemical attractiveness to parasites in creating variation in susceptibility to infection within a host population when individuals share the same environmental conditions. Understanding that is necessary for estimating the role of attractiveness in determining host fitness under parasite‐mediated selection.

We examined the role of host chemical attractiveness to parasites in determining susceptibility to infection using *L. stagnalis* and its trematode parasite *Echinoparyphium aconiatum* (syn. *Pseudechinoparyphium echinatum*) as a model system. We used snail attractiveness to parasite cercariae as a predictor of exposure to them (i.e. contact probability). Additionally, we assessed the roles of host physiological condition (size, food consumption, and respiration rate) and two immune parameters (phenoloxidase [PO]‐like activity and antibacterial activity of hemolymph) in determining the infection process. Our design allowed comparing attractiveness and susceptibility and linking them to host physiological condition and immune function at individual level. Using several measures for host physiological condition also allowed examining the causality of observed effects. We expected high chemical attractiveness to parasites to lead to high susceptibility to infection. However, we assumed high host physiological condition to increase both attractiveness and immune function, thus potentially obscuring the relationship between attractiveness and susceptibility.

## 2 MATERIALS AND METHODS

### 2.1 Study organisms

*Lymnaea stagnalis* inhabits the littoral zone of stagnant and slow‐flowing water bodies across the Holarctic region. It is an important host to numerous parasites, including several castrating trematodes (Erasmus, 1972; Väyrynen, Siddall, Valtonen, & Taskinen, 2000). As natural *L. stagnalis* populations often show low genetic diversity (Kopp, Wolff, & Jokela, 2012), we used snails from a genetically diverse laboratory population in this study. To create this population, we first established seven laboratory stock populations from adult snails collected from seven different ponds in northern Switzerland (see Langeloh, Behrmann‐Godel, & Seppälä, 2017). We then combined 104 adult individuals of the F_2_ laboratory generation from each stock population to form one mixed population. We mass cultured this population for eight generations before the experiment. We maintained all stock populations at 18 ± 3°C and fed the snails with fresh lettuce and Spirulina powder ad libitum.

The parasite *E. aconiatum* has a three‐host life cycle (see Huffman & Fried, 2012). Its definitive host is waterfowl, and it uses *L. stagnalis* as both first and second intermediate host. After sexual reproduction in the bird intestine, parasite eggs are released into the water with feces. From these eggs, free‐swimming miracidia larvae hatch and seek snails. They penetrate the snail gonads and develop into sporocysts that multiply asexually producing rediae. Rediae produce free‐swimming cercaria larvae that seek the next snail host. Among other mechanisms, cercariae locate snails in the environment through chemo‐orientation. They respond to hydrophilic organic molecules (likely possessing amino groups) excreted by the snails (Haas, Körner, Hutterer, Wegner, & Haberl, 1995; Haas, Haberl, et al., 1995). After contact, parasites invade the snail through the urinary orifice and migrate to the hepatopancreas where they encyst as metacercariae. Once the snail gets ingested by a waterfowl, parasites develop to sexual maturity. The parasites used in this study originated from Erlangen‐Höchstadt, Germany (49°39′N/10°49′E). We collected 50 infected adult snails to obtain cercariae. We confirmed the species of released cercariae from each snail through morphological inspection (Faltýnková, Nasincová, & Kablásková, 2007). We maintained these snails in the laboratory as described above for two and a half weeks before the experiment. It is important to note that to our knowledge infecting snails in the role of second intermediate host with trematode cercariae is not reported to lead to as high virulence as infecting them in the role of first intermediate host with miracidia. This is because miracidia develop into parthenitae that castrate the snails and increase their mortality (e.g. Jokela, Lively, Taskinen, & Peters, 1999; Lafferty, 1993; Seppälä, Karvonen, Kuosa, Haataja, & Jokela, 2013). We, however, find *E. aconiatum* cercariae a good model system for investigating infection dynamics in snail–trematode interactions as they allow examining snail susceptibility as a quantitative trait (see below).

### 2.2 Study design

We used 67 adult snails (shell length: 25.7–37.8 mm, mean: 31.3 mm) haphazardly chosen from the stock population to compare their chemical attractiveness to parasite cercariae (a proxy for contact probability with parasites and therefore exposure to infection) as well as susceptibility to infection at an individual level. Additionally, we assessed five explanatory variables to estimate the effects of snail physiological condition and immune function on attractiveness and susceptibility. We measured food consumption and respiration rate (a proxy for metabolic activity) before quantifying attractiveness and susceptibility. Additionally, we measured body size as well as the levels of two immune parameters (PO‐like activity and antibacterial activity) in snail hemolymph at the end of the study (see below for details). We conducted all measurements at 18°C.

### 2.3 Measurements

To quantify food consumption of snails, we placed them individually in cups filled with 200 ml of aged tap water. We soaked fresh lettuce in tap water for 30 min, patted the leaves dry and provided each snail with a weighted (to the nearest 0.01 g) piece that would feed a snail of similar size for at least 2 days under the used conditions. After 24 hr, we removed the remaining lettuce from the cups, patted it dry and recorded the weight. Additionally, we measured the initial and final weight of three pieces of lettuce submerged in water without a snail (i.e. controls) to estimate reduction in weight owing to decomposition. We used the difference in lettuce weight between the beginning and the end of the measurement after subtracting the mean weight loss in controls as an estimate of food consumption.

We measured respiration rate of snails using a Fibox 4 system (PreSens Precision Sensing GmbH, Regensburg, Germany) with optical oxygen sensors. We placed the snails individually in 185 ml vials completely filled with aerated aged tap water. To ensure a homogenous distribution of oxygen within the vials, we constantly stirred the water using magnetic stirrers. After 10 min of acclimation, we measured the oxygen concentration in each vial for 25 times during a 15‐ to 23‐min period. We determined respiration rate (mg O_2_/hr) by calculating the slope of the linear regression line between oxygen level and time and multiplying that by −1.

**Figure 1:**
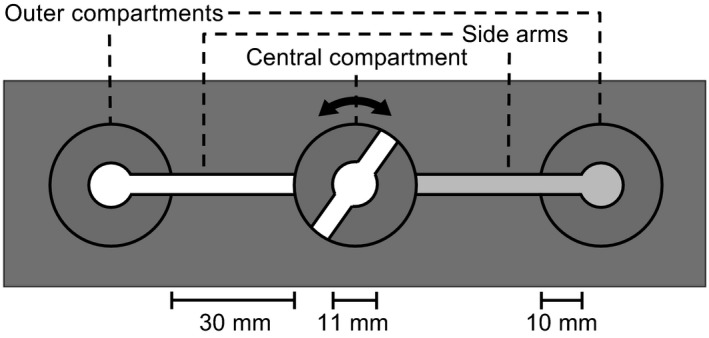
Schematic drawing of a chemo‐orientation test chamber used to measure snail attractiveness to cercariae. The chamber consists of a closable central compartment connected to two outer compartments through side arms. During the assay, the central compartment, one outer compartment, and the connecting side arm were filled with clean water (white) and the other outer compartment and its adjacent side arm with snail‐conditioned water (light gray)

To measure snail attractiveness to parasite cercariae, we used similar test chambers as in Haas, Körner, et al. (1995) and Seppälä and Leicht (2015). Each chamber consisted of three compartments with a closable central compartment that was connected to two outer compartments through side arms on opposite sides (see Figure 1). Before the tests, we produced snail‐conditioned water (SCW) by placing each experimental snail individually in a cup with 100 ml of aged tap water for 2.5 hr (this time period had led to the highest variation in chemo‐orientation responses in pilot tests). We did not feed the snails during this period to ensure that potential compounds dissolving from lettuce could not confound the results. For each assay, we filled the closed central compartment as well as one outer compartment and the connecting side arm with clean aged tap water and the second outer compartment and its adjacent side arm with SCW of the tested snail (which side arm received SCW/clean water was assigned randomly). For each test, we collected fifteen 0‐ to 20‐min‐old cercariae that were a mixture originating from five different infected snails (3 cercariae from each snail). We haphazardly assigned which test chamber received cercariae from which infected snails. This ensured genetic (i.e. clonal) variation in used cercariae both within and between the tests. We introduced the collected cercariae into glass Petri dishes and kept them like that for 60 min before the assays. This was because host finding by freshly emerged cercariae is often reduced to avoid infecting the same snail individual that emitted them (Evans & Gordon, 1983; Haas, Körner, et al., 1995). After that, we placed the cercariae into the closed central compartments of the test chambers. After an acclimation period of 1 min, we opened the central compartments for 3 min. Then, we closed them and counted the number of cercariae in each of the three separated sections of each chamber. The used time period had led to the highest chemo‐orientation responses in pilot tests indicating that a chemical gradient was established in the test chambers.

To measure the susceptibility of snails to infection, we placed each experimental snail individually in a cup with 200 ml of aged tap water. We provided lettuce as a food source for snails. We collected 15 fresh parasite cercariae as described above and introduced them into the cups with the snails. We used an exposure dose of 15 cercariae per snail because it allowed us to examine the susceptibility to infection as a quantitative trait (Seppälä et al., 2011; Wiehn, Kopp, Rezzonico, Karttunen, & Jokela, 2002) and because parasite intensities in the wild commonly range between 10 and 30 metacercariae per snail (pers. obs.). After 24 hr and after measuring size and taking hemolymph samples to quantify immune activity (see below), we removed the snails from their shells, dissected them, and counted the number of encysted metacercariae in snail tissues under a microscope to determine their abundance. The exposure time was sufficient for the parasites to invade the snails and form metacercariae because their infectivity is highest a few hours after the emergence (McCarthy, 1999). The used exposure procedure also leads to high variation in infection rate across snail individuals, which enables examining relationships between snail susceptibility to parasites and other examined traits.

To assess snail body size, we measured their shell length to the nearest 0.1 mm. To quantify immune activity, we measured the levels of two immune parameters, PO‐like activity and antibacterial activity, in snail hemolymph. Phenoloxidase (PO) enzymes are involved in the oxidative defense against mostly eukaryotic parasites (Cerenius & Söderhäll, 2004). Antibacterial activity, on the other hand, reflects the humoral immune response through antimicrobial proteins (Imler & Bulet, 2005). The examined immune parameters are central in the immune system of invertebrates, including mollusks (e.g. Butt & Raftos, 2008; Hellio, Bado‐Nilles, Gagnaire, Renault, & Thomas‐Guyon, 2007; Le Clec'h, Anderson, & Chevalier, 2016; Mitta, Vandenbulcke, & Roch, 2000), and are known to respond to various immune elicitors (Seppälä & Leicht, 2013) as well as to be subject to natural selection in *L. stagnalis* (Langeloh et al., 2017). It is, however, important to note that the role of these traits in determining resistance against *E. aconiatum* is not known. We took the hemolymph samples and measured the immune parameters as described in Seppälä and Jokela (2010). In brief, we collected hemolymph samples by gently tapping the foot of each snail with a pipette tip until it retreated into the shell simultaneously releasing hemolymph through the hemal pore (Sminia, 1981). We measured the PO‐like activity and antibacterial activity of hemolymph spectrophotometrically using a microtiter plate reader (SpectraMax 190, Molecular Devices, Sunnyvale, CA, USA). For the measurements of PO‐like activity, we mixed hemolymph with L‐Dopa and measured the increase in optical density (OD) of the solution followed by an enzymatic reaction in which L‐Dopa is oxidized to dopachrome. For the measurements of antibacterial activity, we mixed hemolymph with lyophilized *Escherichia coli* cells and measured the decrease in OD of the solution followed by the lysis of *E. coli* cells. In case of antibacterial activity, we modified the original protocol so that the measurement took 25 min.

### 2.4 Statistical analyses

To examine whether *E. aconiatum* cercariae showed chemo‐orientation, we analyzed the variation in their preference for the arm of the experimental chamber containing SCW versus clean water. We tested if the proportion of cercariae found in the SCW arm differed from 50% when considering those cercariae that left the central compartment using a one‐sample *t* test. To homogenize error variance, we arcsin(sqrt(*x*))‐transformed these data before the analysis.

We used structural equation modeling in IBM SPSS AMOS 23 (IBM, Armonk, NY, USA) to examine the effects of snail size, food consumption, respiration rate, and the examined immune parameters (PO‐like activity and antibacterial activity of hemolymph) as well as the causality of those effects on the snails' attractiveness to parasite cercariae and their susceptibility to infection. The original model was based on our estimates of the causality of possible impacts and included the following links between the variables: effects of snail size on food consumption (Salo, Stamm, Burdon, Räsänen, & Seppälä, 2017), respiration rate (Salo et al., 2017), attractiveness to parasites, immune function (both parameters (Salo et al., 2017; Seppälä & Jokela, 2010)), and susceptibility to infection (Seppälä et al., 2011); effects of food consumption on attractiveness to parasites (Seppälä & Leicht, 2015), immune activity (both parameters (Langeloh et al., 2017; Seppälä & Jokela, 2010)), and susceptibility to infection (Seppälä et al., 2011); effects of respiration rate on attractiveness to parasites and susceptibility to infection; effects of attractiveness to parasites and the level of immune parameters on susceptibility to infection. Additionally, the model included covariation between food consumption and respiration rate as well as between immune traits (Seppälä & Leicht, 2013). We optimized this model using a model selection procedure in which nonsignificant effects were removed from the model one at a time and the fit of the models was estimated based on chi‐square, root mean square of approximation (RMSEA), comparative fit index (CFI), and Akaike information criteria (AIC) values (see Grace, 2006). When the two compared models did not show large differences in their fit, we chose the simpler model. In this analysis, we estimated the attractiveness of snails to the parasites by calculating the proportion of cercariae in the chamber that were found in the SCW arm. This differs from the measure used in the analysis on chemo‐orientation (see above) as it reflects host's risk to become infected better than the previous measure which estimated host finding from parasite perspective. As a measure of susceptibility to infection, we used the proportion of cercariae successfully infecting the snails when exposed in cups. We arcsin(sqrt(*x*))‐transformed both of these variables before the analysis. As the examined immune traits were removed from the final model (see section results), we analyzed covariation between them separately using a Pearson's correlation.

## 3 RESULTS

**Figure 2:**
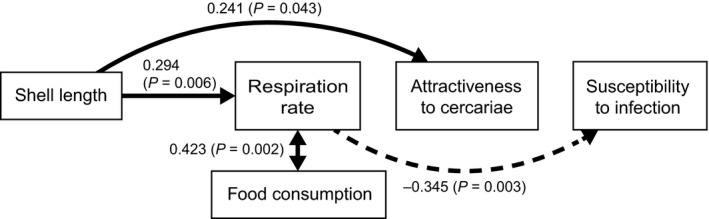
Structural equation model for infection process between *Lymnaea stagnalis* snails and *Echinoparyphium aconiatum* cercariae. Solid arrows indicate positive relationships between variables. Dashed arrows indicate negative relationships. Values next to one‐way arrows refer to standardized regression weights and their significance levels. Values next to a two‐way arrow refer to a correlation and its significance level

**Figure 3:**
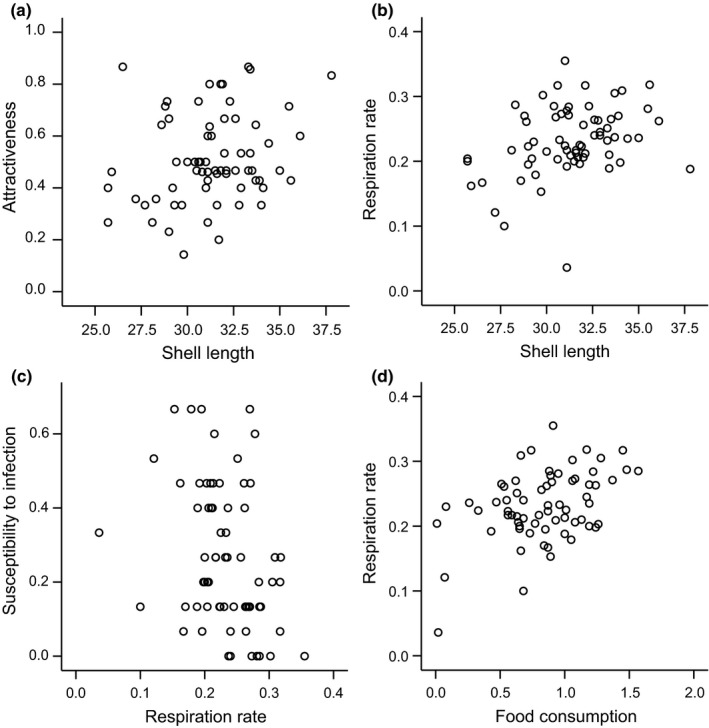
Relationships among variables that were statistically significant in the structural equation model. (a) Shell length (mm) and chemical attractiveness to *Echinoparyphium aconiatum* cercariae (proportion of cercariae found in the snail‐conditioned water arm of the chamber), (b) shell length and respiration rate (mg O_2_/hr), (c) respiration rate and overall susceptibility to *E. aconiatum* infection (proportion of cercariae infecting a snail), and (d) food consumption (g/day) and respiration rate

In the chemo‐orientation test, 70.1 ± 2.5% (mean ± *SE*) of *E. aconiatum* cercariae that left the central compartment of the test chamber moved into the side arm filled with SCW (one‐sample *t* test with a reference of 50%: *t*_66_ = 7.181, *p *<* *0.001). This confirms the capacity of parasites to detect chemical host cues in the environment. Individual snails, however, varied a lot in their attractiveness to cercariae (14%–87% of all cercariae used in chemo‐orientation assays moved toward SCW). Chemical attractiveness to cercariae increased with snail shell length (Figures 2 and 3a), indicating that larger individuals should be exposed to a higher number of parasite transmission stages. When exposed to *E. aconiatum* cercariae, 89.6% of the snails became infected. Similarly to attractiveness, snail susceptibility to infection showed high variation the maximum proportion of cercariae successfully infecting an individual host being 67%. Snail size also contributed to the variation in snail susceptibility, but this effect was negative and indirect through respiration rate (Figure 2). This was because larger snails had higher respiration rates (Figures 2 and 3b) which reduced their susceptibility to infection (Figures 2 and 3c). Likewise, high food consumption indirectly reduced susceptibility to infection through a positive relationship with respiration rate (Figures 2 and 3d). Variation in exposure or susceptibility to parasites was not explained by other variables including the examined immune traits, which, however, were negatively correlated (Pearson's correlation: *N* = 67, *r* = −0.260, *p* = 0.034).

## 4 DISCUSSION

Here, we tested the importance of chemical attractiveness to parasites in determining host susceptibility to infection in a snail–trematode interaction where the parasite has been shown to respond to chemical host cues (Haas, Körner, et al., 1995; Seppälä & Leicht, 2015). We found that attractiveness of *L. stagnalis* snails to *E. aconiatum* cercariae showed high variation across individuals. However, contrary to our expectation, attractiveness to cercariae did not affect host overall susceptibility. This was due to complex effects of snail physiological condition on the infection process. First, attractiveness to cercariae increased with body size. This was most likely due to a higher quantity of chemical cues (e.g. organic molecules (Haas, Körner, et al., 1995)) released by large snails. However, together with high food consumption, large size increased snail respiration rate, which was associated with reduced susceptibility to infection. Therefore, the direct effect of size on snail attractiveness to parasites and its indirect effect on overall susceptibility through metabolic activity overrode each other's effects. Thus, our results suggest that snail characteristics affecting after contact with the parasites are likely to be more important than chemical attractiveness in determining the infection process in this system.

High metabolic activity that reduced snail susceptibility to infection suggests physiological processes including immune function to be important in determining the outcome of infection (e.g. Demas, Chefer, Talan, & Nelson, 1997; Martin, Scheuerlein, & Wikelski, 2003; Svensson, Råberg, Koch, & Hasselquist, 1998). However, the levels of quantified immune parameters, namely PO‐like activity and antibacterial activity of hemolymph, did not explain variation in snail susceptibility. Thus, we cannot determine the mechanism underlying the observed relationship between metabolic rate and susceptibility to infection. It is important to note that to our knowledge, immunological mechanisms determining snail resistance against *E. aconiatum* cercariae are not understood. It is, for example, possible that some localized immune responses in the target tissue of the parasite (i.e. hepatopancreas/digestive gland) determine the infection success and that their levels differ from those in the hemolymph (Tzou et al., 2000; Pulpitel, Pernice, Simpson, & Ponton, 2015; but see Galinier et al., 2013). Localized responses could also be highly beneficial by reducing the energetic costs of immune activation and by limiting self‐harm commonly associated with immune defense (e.g. Graham et al., 2010; Sadd & Siva‐Jothy, 2006). It is, however, important to note that our study investigated the invasion success of the parasites without considering their longer term survival, which could also be affected by the snail's immune system. Furthermore, the examined immune traits did not covary with the attractiveness of snails to parasites indicating a lack of a trade‐off between them. However, antibacterial activity and PO‐like activity were negatively correlated suggesting a trade‐off between these components of the immune system (see also Seppälä & Leicht, 2013). Negative relationships between similar traits are also found in other invertebrates (e.g. Kortet, Rantala, & Hedrick, 2007; Moret & Schmid‐Hempel, 2001; Väänänen, Kortet, & Rantala, 2006), although not in all taxa (see Adamo, 2004; Cotter, Kruuk, & Wilson, 2004).

Contrary to earlier findings, we found no relationship between food consumption and variation in snail attractiveness to parasites. In an earlier study (Seppälä & Leicht, 2015), long‐term, but not short‐term, food deprivation reduced snail chemical attractiveness to *E. aconiatum* cercariae. It is, however, important to note that in this study all snails had been maintained under ad libitum food supply before the study, and thus, their resource levels were probably high. Together, these findings indicate that a strong increase or decrease in resource availability in the environment may be needed to alter excretion of chemical host cues and thus exposure of snails to parasites. Therefore, our results suggest that although variation in resource level, both over space and time, may strongly contribute to infection dynamics in this system (Seppälä et al., 2011), variation in attractiveness among host individuals is unlikely to be of high importance for their relative fitness within populations when all individuals experience similar environmental conditions.

In natural snail populations, the intensity of trematode metacercariae typically increases with host size (e.g. Evans, Whitfield, & Dobson, 1981; Fernández, Hamann, & Kehr, 2013; Flores, Semenas, & Veleizán, 2010; Morley, Lewis, & Adam, 2004). This relationship has been suggested to appear because of variation among host individuals in factors such as attractiveness to cercariae, resistance to infection, and age that defines the time of exposure to parasites (see Evans et al., 1981; Morley et al., 2004). Our study suggests that in *L. stagnalis*–*E. aconiatum* interaction, large size does not predispose snails to infection in a single exposure event. This is because the effects of size on snail attractiveness to parasite cercariae and susceptibility to infection are opposite. This supports the importance of snail age, and thus time to be exposed to parasites, in determining the observed relationships between host size and intensity of trematode metacercariae in nature.

In conclusion, although *L. stagnalis* snails showed high variation in their chemical attractiveness to *E. aconiatum* cercariae, this did not determine their overall susceptibility to infection. This was because large body size increased attractiveness, but also increased metabolic activity which, in turn, reduced susceptibility. Thus, postcontact mechanisms affecting parasite infection success are more likely to determine host susceptibility in this system. However, immunological parameters measured in snail hemolymph showed no association with susceptibility. This suggests that localized, tissue‐specific immune responses might be more important in determining the outcome of infection in our study system. Such mechanisms, however, remain to be investigated and call for tissue‐specific measurements of immune activity.

## CONFLICT OF INTEREST

None declared.

## AUTHOR CONTRIBUTIONS

LL and OS designed and implemented the experiments. OS performed the statistical analyses. LL and OS wrote the manuscript. Both authors read and approved the final manuscript.

## DATA ACCESSIBILITY

Data deposited in the Dryad Digital Repository: https://doi.org/10.5061/dryad.b4m648v.
